# Comparison of guidelines on rectal cancer: exception proves the rule?

**DOI:** 10.1093/gastro/goab034

**Published:** 2021-09-23

**Authors:** Ruoxu Dou, Siqi He, Yanhong Deng, Jianping Wang

**Affiliations:** 1 Department of Colorectal Surgery, The Sixth Affiliated Hospital of Sun Yat-sen University, Guangzhou, Guangdong, P. R. China; 2 Guangdong Provincial Key Laboratory of Colorectal and Pelvic Floor Diseases, The Sixth Affiliated Hospital of Sun Yat-sen University, Guangzhou, Guangdong, P. R. China; 3 Department of Medical Oncology, The Sixth Affiliated Hospital of Sun Yat-sen University, Guangzhou, Guangdong, P. R. China; 4 Department of Gastrointestinal Surgery, Foresea Life Insurance Guangzhou General Hospital, Guangzhou, Guangdong, P. R. China

**Keywords:** clinical practice guideline, rectal cancer, early or locally advanced, comparative study

## Abstract

The standard of care for early or locally advanced rectal cancer is promoted by multiple clinical practice guidelines globally, but the considerable differences between the guidelines may cause confusion. We compared the latest updated clinical practice guidelines from five professional societies/authorities: National Comprehensive Cancer Network, American Society of Colorectal Surgeons, European Society of Medical Oncology, Chinese National Health Commission, and Chinese Society of Clinical Oncology. Key evidence is discussed for a better understanding of some seemingly contradictory recommendations.

## Introduction

Clinical practice guidelines on the management of early or locally advanced rectal cancer have been published extensively across the world. Although the mainstay of the guidelines remains largely consistent, variation in the practice of countries and in the adoption of emerging evidence has posed potential challenge to their readers. To summarize the differences among some of the most-cited guidelines, we compared the National Comprehensive Cancer Network (NCCN) Guidelines for Rectal Cancer (Version 1.2021), the 2020 American Society of Colorectal Surgeons (ASCRS) Clinical Practice Guidelines for the Management of Rectal Cancer [1], the 2017 European Society of Medical Oncology (ESMO) Clinical Practice Guidelines for Rectal Cancer [2], the 2020 Chinese Protocol of Diagnosis and Treatment of Colorectal Cancer by National Health Commission (NHC) [3], and the 2020 Guidelines of Chinese Society of Clinical Oncology (CSCO) for Colorectal Cancer. For a better interpretation of some major differences, related evidence is discussed. Comparisons are categorized as preoperative assessment; surgical, neoadjuvant, and adjuvant treatment; and surveillance in the main text. For the convenience of readers, we list the differences of perioperative assessment and treatment in Table 1 and surveillance in Table 2. The management of metastatic or recurrent rectal cancer is beyond the scope of this article.

**Table 1:** Comparison of the assessment and treatment of patients with rectal cancer among guidelines

Management	NCCN 2020	ASCRS 2020	ESMO 2017	NHC 2020	CSCO 2020
Preoperative assessment					
Definition of the rectum	Below the virtual line from sacral promontory to upper edge of pubic symphysis	≤15 cm from anal verge with body habitus/sex variation	≤15 cm from the anal verge		Various definitions apply, depending on discipline and clinical scenario
Serum biomarker	CEA	CEA	CEA	CEA, CA19-9; AFP and CA125 if related metastasis	CEA, CA19-9
Local staging	MR ± EUS	MR ± EUS	MR ± EUS	MR ± EUS	MR ± EUS
Distant staging	Chest CT, abdomen CT/MR	Chest, abdomen, pelvis CT	Chest, abdomen CT	Chest, abdomen, pelvis CT	Chest, abdomen, pelvis CT
Endoscopy	Colonoscopy + rigid/flexible proctoscopy	Colonoscopy + rigid/flexible proctoscopy	Colonoscopy + rigid proctoscopy	Colonoscopy ± proctoscopy	Colonoscopy
Surgical treatment					
Surgery for pT1 after polypectomy	Unfavorable histology^a^, sessile malignant polyp			Unfavorable histology^a^	Unfavorable histology^a^
Salvage treatment for local excision	TME or adjuvant (chemo)radiation	TME or adjuvant (chemo)radiation	TME	TME	TME or adjuvant (chemo)radiation
T2 or high-risk T1 for local excision	Local excision + pre-/post-operative chemoradiation		Chemoradiation, then local excision		Chemoradiation, then local excision if ycT1
TME distal margin on bowel wall	1–2 cm	Above anorectal ring: 2 cm; below anorectal ring: 1 cm		>5 cm from the anal verge: 2 cm; ≤5 cm from the anal verge: 1 cm	
TME distal margin on mesorectum	4–5 cm for tumor located above peritoneal reflection	5 cm for tumor located above peritoneal reflection	5 cm for tumor located above peritoneal reflection	5 cm for tumor located above peritoneal reflection	
Laparoscopic TME	Consider experience; contraindicated in: MRF+, obstruction, perforation	Consider experience	Consider experience, body habitus, previous surgery	Consider experience and other factors	Consider experience; contraindicated in obstruction
Lymphadenectomy + ligation at root of IMA	Only for clinically positive LN	Only if clinically positive LN or needed for tension-free anastomosis			Routinely recommended
Lateral pelvic LN dissection	Only for clinically positive LN	Only for clinically positive LN	Only for enlarged LN persistent after chemoradiation	Only for clinically positive LN	Only for clinically positive LN
Neoadjuvant therapy					
Interval to TME	LCCRT: 5–12 weeks; SCRT: ≤1 or 4–8 weeks		LCCRT: 4–12 weeks; SCRT: ≤1 or 4–12 weeks	LCCRT: 5–12 weeks; SCRT: ≤1 or 6–8 weeks	LCCRT: 5–12 weeks
LCCRT	T3 with MRF-; T1–2N1–2	Stage II–III	T3c/d, very low, TD+, or EMVI+ tumors with MRF-; MRF+/T4/lateral LN+	T3c/d, very low, TD+, or EMVI+ tumors with margin-; MRF+/T4/lateral LN+	1st choice for all T3/4 or N+
SCRT	T3 with MRF-; T1–2N1–2. MDT discussion required	Stage II–III	T3c/d, very low, TD+, or EMVI+ tumors with MRF-. Delay surgery for frailty	T3c/d, very low, TD+, or EMVI+ tumors with margin-	2nd choice for T3N0
TNT	MRF+, T4, unresectable; T3 with MRF-; T1–2N1–2; FOLFOXIRI for T4N+	Chemotherapy before/after LCCRT for ≤6 months	SCRT + consolidation for MRF+/T4/lateral LN+		
Watch and wait if cCR for patient unfit or rejecting TME	Stage II–III	Stage II–III	Stage II–III except for MRF+/T4/lateral LN+	Stage II–III	Stage II–III
Chemotherapy without radiation	Investigational, except for upper 1/3 T3N0 MRF-	Optional radiation after chemotherapy	Investigational	Optional radiation after chemotherapy for T3/N+	
Upfront TME	T1–2N0; low-risk upper 1/3 T3N0	T1–2N1; upper 2/3 T ≤ 3N0; ≥10 cm from anal verge	T1–2N0; upper 2/3 T3a/bN0–1 TD- EMVI-; ≥12 cm from anal verge	T2N0; ≥12 cm from anal verge	T2N0
Adjuvant therapy					
Interval after TME	As soon as medically able	≤8 weeks			As soon as medically able; ≤8 weeks
After Neoadjuvant and TME	Perioperative therapy ≤6 months; FOLFOX for 4 m after LCCRT; not required after TNT	Doublet chemotherapy for 4 months, even for pCR	Only for yp stage III or high-risk yp stage II	Only for yp stage III or pMMR high-risk yp stage II	Perioperative therapy ≤6 months
After upfront TME	T3N0: doublet/observe; T4/N+: doublet + radiation	Chemoradiation for CRM+, perforation, T4/N1c/N2; chemotherapy for others	Chemoradiation for CRM+, perforation, T4, N1c/N2 with mesorectal defect	Chemoradiation for CRM+, T4b, N2 with mesorectal defect, PNI close to MRF	Chemoradiation for T3–4 or N1–2

NCCN, National Comprehensive Cancer Network; ASCRS, American Society of Colorectal Surgeons; ESMO, European Society of Medical Oncology; NHC, National Health Committee; CSCO, Chinese Society of Clinical Oncology; CEA, carcinoembryonic antigen; CA, carcinoantigen; CT, computed tomography; MR, magnetic resonance; EUS, endorectal ultrasound; TME, total mesorectal excision; cCR, clinical complete remission; MRF, mesorectal fascia; LN, lymph node; IMA, inferior mesenteric artery; LCCRT, long-course chemoradiotherapy; SCRT, short-course radiotherapy; EMVI, extramural vascular invasion; TD, tumor deposit; MDT, multidisciplinary treatment; TNT, total neoadjuvant therapy; PNI, perineural invasion.

a Unfavorable histology includes grade 3 or 4, angiolymphatic invasion, positive or uncertain margin, fragmented specimen, or tumor budding.

**Table 2.** Comparison of surveillance of patients with rectal cancer among guidelines

Surveillance	NCCN 2020	ASCRS 2021^a^	ESMO 2017	NHC 2020	CSCO 2020
History and physical	Stage II–IV: every 3–6 m for 2 y, then every 6 m for 3 y	Every 3–12 m for 2 y, then every 6–12 m for 3 y	Every 6 m for 2 y	Every 3 m for 2 y, then every 6 m for 3 y, then annually	Stage I: every 6 m for 5 y. Stage II–IV: every 3 m for 3 y, then every 6 m for 2 y, then annually
CEA	Stage II–IV: every 3–6 m for 2 y, then every 6 m for 3 y	Every 3–12 m for 2 y, then every 6–12 m for 3 y	Every 6 m for 3 y	CEA and CA19-9: every 3 m for 2 y, then every 6 m for 3 y	Stage I: every 6 m for 5 y. Stage II–IV: every 3 m for 3 y, then every 6 m for 2 y, then annually
CAP CT	Stage II–III: every 6–12 m for 5 y. Stage IV: every 3–6 m for 2 y, then every 6–12 m for 3 y	2 times in 5 y or up to annually for 5 y	≥2 in the first 3 y	Every 6 m for 2 y, then annually for 3 y	Stage III: annually. Stage IV: every 6–12 m
Colonoscopy	1 y after surgery; 3–6 m if no preoperative colonoscopy. Repeat in 1 y if advanced adenoma^b^; repeat in 3 y, then 5 y if none	1 y after surgery; 1–6 m if no preoperative colonoscopy. Repeat in 3–5 y if no finding	Every 5 y to age of 75. Within 6 m after surgery if no preoperative colonoscopy	1 y after surgery; 3–6 m if no preoperative colonoscopy. Repeat in 1 y if polyp; repeat in 3 y, then 5 y if none	1 y after surgery; 3–6 m if no preoperative colonoscopy. Repeat in 1 y if advanced adenoma^b^; repeat in 3 y, then 5 y if none
Pelvic MR				If CT contraindicated: CAP MR every 6 m for 2 y, then annually for 3 y	Stage III: annually. Stage IV: every 6–12 m
Proctoscopy	For local excision: every 3–6 m for 2 y, then every 6 m for 3 y; + MR/EUS	Anastomosis: every 6–12 m; local excision: every 6 m; for 3–5 y; ±EUS			
PET/CT	Not recommended, consider if CEA elevated but CT-	Not recommended unless CT contraindicated or +	Not recommended, consider if recurrence+	Not recommended, consider if recurrence/metastasis±	Not recommended, consider if CEA elevated but CT-

NCCN, National Comprehensive Cancer Network; ASCRS, American Society of Colorectal Surgeons; ESMO, European Society of Medical Oncology; NHC, National Health Committee; CSCO, Chinese Society of Clinical Oncology; m, month(s); y, year(s); CEA, carcinoembryonic antigen; CA, carcinoantigen; CAP, chest, abdomen, and pelvis; CT, computed tomography; MR, magnetic resonance; EUS, endorectal ultrasound; PET, positron emission tomography.

^a^ For patients with rectal cancer with high-risk stage I, stage II and III, or stage IV treated with curative intent.

^b^ Villous polyp, polyp >1 cm, or high-grade dysplasia.

Where controversies among guidelines are present, although it is tantalizing to judge the superiority of guidelines, this urge is refrained by the authors for two reasons. First, high-level evidence is often still lacking or accumulating in these subjects. Second, available study may be limited by design or setting that restricts its generalizability. Therefore, when disparity is encountered, we try to describe the most relevant studies with their strengths and limitations, so that readers may decide for themselves which guidelines to apply in various clinical settings.

## Preoperative assessment

### Definition of the rectum

The ESMO guidelines define tumors with a distal extension of ≤15 cm from the anal verge as rectal cancer and more proximal tumors as colonic cancer, and further classify rectal cancers into low (≤5 cm), middle (from >5 to 10 cm), or high (from >10 to 15 cm). The ASCRS guidelines shared the 15-cm definition with variation by body habitus and sex, after a discussion on the limitation of various anatomical landmarks such as the upper boundary of the rectum. The NCCN guidelines for rectal cancer define the rectum as the intestine below the virtual line from the sacral promontory to the upper edge of the pubic symphysis. While the NHC guidelines have not defined the limits of the rectum, the CSCO guidelines acknowledge various definitions, depending on the discipline and clinical scenario.

### Serum biomarkers

All guidelines endorse the serum biomarker carcinoembryonic antigen (CEA) for the initial evaluation and follow-up of rectal cancer. The NHC and CSCO guidelines also recommend carbohydrate antigen (CA) 19–9, which is not mentioned in the NCCN or ESMO guidelines. The ASCRS guidelines withhold support for CA 19–9 due to insufficient evidence to support routine use, based on the ASCO 2006 updates of recommendations [4]. The NHC guidelines also recommend alpha fetoprotein (AFP) for patients with suspected liver metastasis and CA 125 for patients with suspected peritoneal or ovarian metastasis. The potential prognostic value of CA 19–9 has been reported in the Asian population in a recent meta-analysis [5] and is worth further evaluation.

### Staging

For tumor (T) and lymph-node (N) staging, pelvic magnetic resonance (MR) is recommended as the routine workup by NCCN, ASCRS, ESMO, NHC, and CSCO guidelines. Other prognostic and treatment-modifying parameters in a structured MR report include mesorectal fascia (MRF) involvement and extramural vascular invasion (EMVI) [6]. Specifically for T3 lesions, the ASCRS, ESMO, and Chinese guidelines consider an MR-reported extramural tumor depth of >5 mm as a high-risk feature indicating neoadjuvant therapy, which is not mentioned in the NCCN guidelines.

All guidelines consider endorectal ultrasound (EUS) complementary to MR by the potential added value of distinguishing T1 vs T2 lesions. With its limitation for MRF and N staging, EUS is not recommend as a substitute for pelvic MR, even for early rectal cancers.

All guidelines support chest computed tomography (CT) and abdominal CT or MR for the detection of metastasis. If feasible, colonoscopy is recommended before surgery to rule out synchronous malignancy.

## Surgical treatment

### Endoscopic polypectomy

The NCCN guidelines define an endoscopically removed polyp as “malignant” when submucosa is invaded. For pT1 lesions without unfavorable histologic features (grade 3 or 4, angiolymphatic invasion, positive or uncertain margin, fragmented specimen, and tumor budding), further local excision or transabdominal resection is not indicated. For a sessile malignant polyp, however, the NCCN guidelines have included additional surgery as well as observation due to a higher likelihood of adverse outcomes. In contrast, the NHC and CSCO guidelines consider additional surgery unnecessary in this scenario. Of note, the NHC guidelines require exclusion of nodal involvement with imaging as a prerequisite for endoscopic removal of the malignant polyp.

### Local excision

In all guidelines, local excision is indicated for cT1N0 cancers without adverse features (grade 3 or 4, lymphovascular invasion, and perineural invasion). Both the NCCN and ESMO guidelines endorse transanal endoscopic microsurgery with potentially better oncologic control, especially for more proximal lesions. In the case of unfavorable pathological findings after local excision, the ESMO and NHC guidelines recommend total mesorectal excision (TME) as the standard salvage option, whereas the NCCN, ASCRS, and CSCO guidelines include adjuvant (chemo)radiation as an alternative. Unfavorable pathological findings include poor differentiation, tumor budding, or lymphovascular or perineural invasion in most guidelines, although the ESMO guidelines consider sm ≥2 (submucosal invasion to the middle or lower third) as an adverse pathological feature compared with sm3 (submucosal invasion to the lower third) in other guidelines.

For selective T2 or high-risk T1 patients who prioritize sphincter preservation or those who are unfit for radical resection, local excision with preoperative chemoradiation has been compared with standard resection in clinical trials with comparable outcomes [7, 8]. The ASCRS and the ESMO guidelines mention this option as an alternative under investigation, preferably in clinical trials. The CSCO guidelines also propose neoadjuvant chemoradiation for cT2N0 patients as a secondary alternative, and further stratify cases with clinical complete remission (cCR) to watch and wait, ycT1 to local excision, and ycT2 to radical resection. This alternative strategy to radical resection for T2 or high-risk T1 is not endorsed by the NCCN or the NHC guidelines.

### TME

TME is recommended as the standard radical resection by all guidelines, although slightly different resection margins are applied. For low rectal cancer, a distal resection margin with a 1- to 2-cm bowel wall is recommended by the NCCN guidelines. The ASCRS guidelines suggest a 2cm bowel wall for tumors above the anorectal ring and a 1-cm bowel wall below it. Similarly, the NHC guidelines require 1 cm for tumors within 5 cm of the anal verge and 2 cm for tumors >5 cm from the anal verge.

The compared guidelines have similar requirements for the distal mesorectal resection margin. For tumors with a lower border below the peritoneal reflection, TME, i.e. the complete removal of the mesorectum to the level of the anorectal ring, is typically recommended. For tumors above the peritoneal reflection, a tumor-specific mesorectal excision with a 5-cm distal mesorectal margin is proposed by the ASCRS, ESMO, and NHC guidelines. The NCCN guidelines allow a slightly more tolerant 4–5 cm.

Despite the publications of several randomized studies including COLOR II [9], CLASICC [10], COREAN [11], ACOSOG Z6051 [12], and ALaCaRT [13], the NCCN and CSCO guidelines remain inconclusive on the oncologic outcomes of laparoscopic resection, and therefore reserve the approach for experienced surgeons or centers. The NCCN guidelines further specify that laparoscopy is contraindicated in patients with MRF involvement, obstruction, or perforation. In comparison, the ESMO guidelines include surgeon experience, patient habitus, and previous open abdominal surgery in the consideration of laparoscopy vs open surgery. The ASCRS guidelines take a more affirmative stance for laparoscopy by changing the statement grade from 1B in 2013 to 1 A. Robotic-assisted surgery is considered under evaluation by most guidelines. Similarly, although transanal TME may facilitate low rectal mobilization in difficult situations, its oncological outcome awaits further assessment.

### Extended lymph-node dissection

The ASCRS guidelines specify the level of vascular ligation and lymphadenectomy at the origin of the superior rectal artery (low tie) as compared with ligating the inferior mesenteric artery (IMA) and dissection of associated lymphatic tissue at its takeoff from the aorta (high tie). High tie is indicated when lymphadenopathy is suspected at the root of the IMA (station 253) and when additional mobilization is needed for tension-free anastomosis. With insufficient evidence to support or refute the routine practice of high tie, the guideline grade for low tie is adjusted from 1 A in 2013 to 1B. The NCCN guidelines do not recommend extended lymph-node dissection as routine practice, but also state that clinically suspicious nodes beyond the field of resection should be biopsied and/or removed if possible. However, NCCN does not specify whether station 253 is included in the field of routine resection. In comparison, the CSCO guidelines require dissection at the root of the IMA as an integral part of radical resection.

Similarly, the ASCRS and CSCO guidelines do not recommend lateral pelvic lymph-node dissection (LPLND) in the absence of a “clinically positive” lymph node. The ESMO guidelines categorize a positive lateral node as the “advanced (ugly)” risk group and recommend preoperative chemoradiation; LPLND is indicated only for enlarged lateral nodes persisting after chemoradiation. None of the guidelines defines the optimal criterion of positive lateral nodes with or without chemoradiation, although the ASCRS guidelines cite studies that consider positive lateral nodes with a short axis of 7 or 5 mm as the cut-off [14, 15].

## Neoadjuvant therapy

### Standard schedules for neoadjuvant (chemo)radiation and interval to surgery

Neoadjuvant therapy with TME is considered the standard treatment for locally advanced mid/low rectal cancer. The ASCRS guidelines recommend neoadjuvant therapy typically for stage II/III rectal cancer. Current evidence shows that preoperative radiation reduces local recurrence without improvement in overall survival. Two different schedules are accepted by all guidelines as the standard of care: long-course chemoradiotherapy (LCCRT) and short-course radiotherapy (SCRT). LCCRT consists of 1.8–2 Gy per fraction over 5–5.5 weeks for a total dose of 45–50.4 Gy. This is followed by a 5- to 12- (NCCN, NHC, and CSCO) or 4- to 12-week (ESMO) interval before surgery. SCRT with 5 Gy daily for 5 days is an alternative regimen, with immediate (within a week) or delayed surgery (NCCN, 4–8 weeks; ESMO, 4–12 weeks; NHC, 6–8 weeks) after the last radiation.

### Indication for neoadjuvant (chemo)radiation

Compared with SCRT with immediate surgery, LCCRT (with delayed surgery) is considered more effective in inducing pathologic downstaging. As a result, the CSCO guidelines recommend LCCRT for all T3/4 or N+ cancers as the first choice and SCRT as a second choice only for T3N0 lesions. However, the Stockholm III trial randomly assigned patients with rectal cancer to SCRT with immediate surgery, SCRT with delay, or LCCRT with delay, and showed similar OS at a median follow-up of 5 years [16]. Of note, the SCRT-delay group had a higher rate of pathological complete response (pCR, 11.8% vs 1.7%, *P *=* *0.001) and a lower risk of post-operative complications (41% vs 53%, *P *=* *0.001). The recently published RAPIDO trial randomly assigned 920 patients with locally advanced rectal cancer to SCRT with consolidation chemotherapy or LCCRT, and found fewer 3-year disease-related treatment failure in the SCRT group (23.7% vs 30.4%, *P *=* *0.019) [17]. Accordingly, the ESMO guidelines and NHC guidelines recommend either LCCRT or SCRT for T3c/d, very low, tumor deposit–positive, or EMVI-positive tumors with resection margin not at risk (the bad risk group). For the advanced (ugly) risk group (MRF+/T4/lateral node+), both guidelines advise SCRT with consolidation chemotherapy or LCCRT to achieve maximal tumor regression; for elderly or frail patients, SCRT with delayed surgery can be considered. Based on updated literature, the NCCN guidelines also lifted their restriction of SCRT for patients with T3 rectal cancer in previous versions, while emphasizing multidisciplinary evaluation of long-term toxicity and the need for downstaging for this approach.

### Concurrent chemotherapy with neoadjuvant radiation

All guidelines recommend continuous intravenous 5-fluorouracil infusion or oral capecitabine during LCCRT. For SCRT, no concurrent chemotherapy is needed. The addition of oxaliplatin to the standard 5-fluorouracil or capecitabine sensitized LCCRT has been studied by six large randomized trials (ACCORD 12 [18], STAR-01 [19], R-04 [20], CAO/ARO/AIO-04 [21], FOWARC [22], and PETACC6 [23]). With increased toxicity, the results showed an inconsistent benefit of pCR rates among studies. In four out of five trials with long-term outcome, survival was not improved with the addition of oxaliplatin. Similarly, adding targeted agents concurrently to LCCRT did not show significant improvement.

### Sequential chemotherapy with neoadjuvant radiation

The sequential addition of chemotherapy before (induction) or after (consolidation) neoadjuvant radiation, also referred to as total neoadjuvant therapy (TNT), has been shown to improve oncological control. The mechanism may involve early treatment of micrometastases and better completion rates of perioperative chemotherapy. For T3 with uninvolved MRF or T1–2N1–2 tumors, the NCCN guidelines add TNT as an alternative to LCCRT or SCRT. For MRF+, T4, surgically unresectable, or medically inoperable tumors, the NCCN guidelines recommend TNT as the standard treatment. With the publication of the PRODIGE 23 results [24], the FOLFOXIRI triplet regimen will be increasingly considered for T4N+ lesions. The ASCRS guidelines also mention TNT with a duration of no more than 6 months and highlight sequential chemotherapy before or after LCCRT rather than SCRT. As mentioned earlier, the ESMO guidelines and NHC guidelines advise SCRT with consolidation chemotherapy (the RAPIDO trial [17]) or LCCRT for the advanced (ugly) risk group (MRF+/T4/lateral node+) to achieve maximal tumor regression.

Until now, limited data have been available for the comparison of induction vs consolidation chemotherapy. The CAO/ARO/AIO-12 study shows higher pCR for consolidation (25%) vs induction (17%) chemotherapy, but the long-term oncological outcome is pending [21].

### Watch and wait

For stage II/III rectal cancer restaged as cCR after (total) neoadjuvant therapy, the NCCN guidelines recommend transabdominal resection as the standard treatment. However, a watch-and-wait non-operative approach may be considered in centers with experienced multidisciplinary teams, after a careful discussion about risk tolerance with the patient. The ASCRS guidelines believe that these patients should typically be offered radical resection, while a watch-and-wait approach can be considered for highly selected patients (especially if radical resection would jeopardize sphincter preservation) with a predefined follow-up protocol. The NHC and CSCO guidelines have similar recommendations. In contrast, the ESMO guidelines withhold the watch-and-wait approach from the advanced (ugly) risk group.

The current proposed criteria for cCR typically include no palpable tumor at DRE, no visible lesion other than a flat scar at endoscopy, ycT0N0 at restaging MR, and a normal CEA level. The guidelines agree in that there is discrepancy between cCR and pCR, and that a more frequent follow-up protocol with MR is recommended. For lack of established selection criteria and large prospective studies with long-term outcome, watch and wait currently remains an investigational approach.

### Neoadjuvant chemotherapy without radiation

For T3 tumors not threatening the MRF or T4a tumors in the mid and upper rectum, a doublet chemotherapy (FOLFOX or CapeOX) with omission of radiation may spare patients from the associated morbidities without compromising oncological control [22, 25]. However, limited long-term data are available for tumors with higher risk (MRF+, etc.) and the result of the PROSPECT trial is pending [26]. The NCCN guidelines consider this approach still investigational for most stage II/III rectal cancer, except for T3N0, margin-negative tumors high in the rectum. Likewise, the ESMO guidelines do not recommend this approach outside clinical trials. In comparison, the NHC guidelines typically recommend neoadjuvant chemoradiotherapy for T3 or N+ patients, but allow initial neoadjuvant chemotherapy after multidisciplinary discussion and optional radiation based on response to chemotherapy. The ASCRS guidelines also acknowledge the option to consider selective radiotherapy depending on tumor response after induction chemotherapy.

### Upfront surgery without neoadjuvant therapy

Upfront radical resection can be the standard treatment for adequately staged early rectal cancer. The NCCN guidelines support this option for T1–2N0 and low-risk upper T3N0 tumors. The ASCRS guidelines also mention selective omission of neoadjuvant therapy for patients with T ≤ 3N0 tumor ≥5 cm from the anal verge or T1–2N1 tumor. The ASCRS guidelines further cite the MERCURY [27] and QuickSilver [28] trials, which propose upfront surgery on patients with MR-predicted circumferential resection margin (CRM)-, EMVI-, and T3a/b (extramural spread <5 mm) tumors. The ESMO guidelines specifically summarize the indication for upfront TME as the “early (good)” risk group, which includes cT1–2N0 and (for the middle or upper rectum) cT3a/bN0–1 without tumor deposit or EMVI. The NHC and CSCO guidelines share a more conservative recommendation of upfront surgery only in T2N0 tumors.

Of note, neoadjuvant chemoradiation is not indicated for upper rectal cancers. The ASCRS and CSCO guidelines draw the cut-off at 10 cm from the anal verge, whereas the ESMO and NHC guidelines have the line at 12 cm from the anal verge.

## Adjuvant therapy

### Interval to adjuvant therapy

A meta-analysis of 15,410 patients with stage II/III colorectal cancer in 2011 showed a 14% decrease in overall survival and disease-free survival with every 4-week delay of adjuvant therapy [29]. This is corroborated by an updated systemic review in 2016 that also demonstrated the negative impact of a delay of >8 weeks [30]. Hence, for patients indicated for adjuvant therapy, the NCCN guidelines recommend the initiation of treatment as soon as the patient is medically able. The ASCRS and CSCO guidelines further specify an interval of no more than 8 weeks after surgery.

### Adjuvant therapy after neoadjuvant therapy and TME

Compared with the established role in colon cancer, high-quality evidence of adjuvant chemotherapy in rectal cancer has been lacking, with a potential confounding impact from neoadjuvant chemoradiotherapy. Several randomized trials (EORTC 22921 [31], I-CNR-RT [32], and DCCG [33]) have failed to demonstrate the benefit of adjuvant fluoropyrimidine monotherapy after neoadjuvant therapy. The controversy has been focusing on the role of adjuvant doublet therapy thereafter [34].

The ADORE trial compared adjuvant FOLFOX with fluorouracil plus leucovorin in patients with yp stage II/III rectal cancer after neoadjuvant LCCRT and TME, and found improved overall survival and disease-free survival in the FOLFOX group [35]. The benefit was not statistically significant in yp stage II patients, suggesting patient selection in this subgroup [35]. Accordingly, the ESMO guidelines consider adjuvant chemotherapy as reasonable practice only for patients with yp stage III or high-risk yp stage II tumors. Similarly, the NHC guidelines recommend adjuvant chemotherapy for yp stage III tumors and high-risk yp stage II pMMR tumors, regardless of whether neoadjuvant therapy is given. Of note, the aforementioned CAO/ARO/AIO-04 and PETACC6 have investigated the addition of oxaliplatin in both neoadjuvant and adjuvant therapies. However, the benefit of adjuvant doublet is probably confounded by the neoadjuvant therapy due to the study design [23, 36].

Nevertheless, retrospective studies have shown the benefit of adjuvant chemotherapy even in patients with pCR, supporting a generalized strategy [37, 38]. For patients with clinical stage II or III tumors who were treated with neoadjuvant therapy, the ASCRS guidelines typically recommend adjuvant doublet chemotherapy regardless of the final pathological stage, including pCR. The NCCN guidelines recommend perioperative therapy for a total duration of ≤6 months; a 4-month course of FOLFOX is typically recommended if neoadjuvant LCCRT has been given. The CSCO guidelines also recommend adjuvant chemotherapy regardless of pathological stage for a total of 6 months of perioperative therapy.

### Adjuvant therapy after upfront surgery

For patients with rectal cancer treated with upfront resection, the NCCN guidelines recommend observation for pT1–2N0M0 tumors and pT3N0M0 tumors in the upper rectum with favorable histologic features (grade 1 or 2, invading <2 mm into the perirectal fat, without lymphatic or venous vessel involvement) [39]. Doublet chemotherapy is recommended for other pT3N0 tumors, whereas doublet chemotherapy with sequential chemoradiation is recommended for pT4 or N+ tumors. In comparison, for patients with pathologic stage II or III tumors receiving upfront resection, the ASCRS guidelines typically recommend adjuvant chemotherapy, whereas adjuvant chemoradiotherapy is recommended only in high-risk patients, characterized by CRM+, perforation, or T4/N1c/N2. Similarly, the ESMO, NHC, and CSCO guidelines also recommend adjuvant chemoradiation for patients with adverse histopathological features after upfront surgery. Of note, all guidelines point out the inferior survival of upfront surgery and post-operative chemoradiation in patients indicated for neoadjuvant chemoradiation, underscoring the importance of adequate preoperative assessment and multidisciplinary decision-making.

### Surveillance

Surveillance of patients after curative treatment of rectal cancer is described in all except the ASCRS guidelines, instead of which the 2021 ASCRS guidelines for surveillance are included for comparison [40]. The ASCRS, ESMO, and NHC guidelines each propose a relatively uniform follow-up strategy for most stages of the disease, whereas the NCCN and CSCO guidelines endorse a more stage-specific approach. Despite the differences in modalities and time points, clinical examination, CEA, CT, and colonoscopy are commonly applied across all guidelines, whereas MR and proctoscopy are inconsistently recommended. Positron emission tomography is not recommended by any guidelines for surveillance purposes, unless recurrence or metastasis is clinically suspected.

## Conclusions

As the saying goes, exception proves the rule. Although some of the differences among guidelines represent the historical practice of countries and regions, most differences actually reflect controversies over lack of evidence or disparities in the appraisal of emerging evidence. This is natural considering that the school of colorectal surgery continues to evolve. Current treatment modalities are constantly being challenged and updated by new concepts or techniques, to provide better oncological control, functional outcome, and cost-effectiveness for our patients. This article is intended not only to summarize differences for clinical reference, but also to analyse their background and shed light on potential research opportunities.

## Authors’ Contributions

Conception and design: R.D., J.W. Writing and revision of the manuscript: R.D., S.H. Review of the manuscript: Y.D., J.W.

## Funding

This work was supported by the Sixth Affiliated Hospital of Sun Yat-Sen University Clinical Research – “1010” Program [1010PY (2020)–12] to R.D.

## Conflict of Interest

None declared.
